# Rubella antibody levels in the healthy Chinese population: a meta-analysis

**DOI:** 10.3389/fimmu.2024.1472189

**Published:** 2024-11-12

**Authors:** Yaning Zhuo, Zhaojun Lu, Xuechao Zhang, Xiaoping Zhang, Yingying Yang, Jiayin Han, Jian Du, Yuyang Xu, Yan Liu, Chuanxi Fu

**Affiliations:** ^1^ School of Public Health, Zhejiang Chinese Medical University, Hangzhou, China; ^2^ Department of Expanded Program on Immunization, Hangzhou Center for Disease Control and Prevention, Hangzhou, Zhejiang, China; ^3^ The Institute of Infectious Disease and Vaccine, School of Public Health, Zhejiang Chinese Medical University, Hangzhou, China

**Keywords:** rubella, IgG antibody, vaccination, Chinese population, meta-analysis

## Abstract

**Objectives:**

To gain a comprehensive understanding of rubella seroprevalence in the healthy population in China and to offer data-driven support for the goal of rubella elimination.

**Methods:**

CNKI, Wanfang database, VIP database, CBM, PubMed, web of Science, and Embase databases were searched to collect studies reporting the positive rate of rubella antibody among the Chinese healthy populations between 2001 and 2022. We conducted a meta-analysis using R language, and data were combined using random effects model.

**Results:**

A total of 97 studies were included, encompassing a sample size of 103,018. Meta-analysis showed that the overall prevalence of rubella antibody positivity in the Chinese healthy population was 77.29% (95% CI: 75.26-79.33). The prevalence of rubella antibody varied significantly based on age and vaccination status. Among the age groups studied, the <1-year-old group exhibited the lowest positivity rate for rubella antibody at 47.87% (95% CI: 41.53-54.21),while the ≥50 years old group showed the highest positivity rate at 85.43% (95% CI: 81.01-89.85); individuals with a history of vaccination demonstrated a higher antibody positivity rate compared to those without vaccination history.

**Conclusions:**

The prevalence of rubella antibody in healthy Chinese population is relatively low. In order to establish a solid immunity threshold levels, it requires conducting timely immunization programs on key groups such as middle school students and the women of childbearing age on the basis of ensuring a high level of rubella-containing-vaccines (RCV) coverage, so as to achieve the goal of eliminating rubella.

**Systematic review registration:**

https://www.crd.york.ac.uk/PROSPERO/, identifier CRD42024607949.

## 1 Introduction

Rubella is an acute infectious disease caused by the rubella virus, primarily transmitted through respiratory droplets and direct contact. The main clinical manifestations of rubella include fever, a generalized erythematous maculopapular rash, and lymphadenopathy. Complications may include arthralgia, arthritis, thrombocytopenic purpura, and encephalitis (1). While the general clinical symptoms of rubella are usually mild, the infection with the rubella virus during early pregnancy can lead to adverse outcomes such as spontaneous abortion, stillbirth, or congenital rubella syndrome (CRS) in newborns. Currently, there is no specific clinical treatment for CRS. The World Health Organization (WHO) estimates that approximately 100,000 cases of CRS occur worldwide annually, posing significant challenges to the advancement of public health. However, these adverse consequences of rubella can be prevented and controlled through the administration of RCV, potentially leading to eventual elimination of rubella (2).

In 1993, China introduced the rubella vaccine (RV); however, its availability for vaccination is limited to certain regions (3). In 2008, China included measles-rubella vaccine (MR) and measles-mumps-rubella vaccine (MMR) into the national expanded program on immunization, implementing a two-dose immunization schedule for eligible children (4). The coverage of RCV immunization has steadily increased and has consistently remained above 95% since 2012. The incidence of rubella in China decreased from 9.11/100,000 in 2008 to 0.12/100,000 in 2017. Nevertheless, there was a notable increase in rubella incidence during the years 2018 to 2019 (5). In this study, we employed meta-analysis to investigate the nationwide rubella seroprevalence utilizing data from rubella antibody surveillance among healthy populations conducted in various regions of China over multiple years, aiming to enhance rubella immunization strategies.

## 2 Methods

### 2.1 Data searches

Literature to be included in the meta-analysis was sought in the Chinese data base and English data base respectively. Chinese data bases included CNKI, Wanfang database, VIP database and CBM; English data bases included PubMed, Web of Science, Embase. The searching terms were (Rubella OR Rubellas OR Three Day Measles OR Three Day Measle OR German Measles) AND (Antibody positive rate OR seropositive OR seropositivity OR serosurvey OR serosurveillance OR seroprevalence) AND (Enzyme-linked immunosorbent assay OR ELISA) AND (China OR Chinese). The search period for this study spanned from the inception of the database up to December 31, 2023.

### 2.2 Literature selection

①The study population consisted of physically healthy individuals; ②Seroprevalence were examined using enzyme-linked immunosorbent assay (ELISA), values≥20 IU/ml were considered positive, values of 10-20 IU/ml were considered equivocal, and values<10 IU/ml were considered negative; ③The positive rates were either directly reported or had data available for calculating the positivity rates.

### 2.3 Data extraction and quality assessment

Two investigators independently evaluated the abstracts of the literature independently and screened them according to the literature selection and exclusion. Then read the full articles, which were scored based on the quality assessment criteria. When encountering differences, the investigators can consult with each other or seek resolution from a third investigator to reach a final decision regarding inclusion. After completing the literature screening process, data including the first author, publication year, age, gender, survey time, survey location, number of positive antibody individuals, sample size, antibody positive rate and immunization history were extracted from the articles.

The literature using the assessment criteria of the cross-sectional studies which recommended by Joanna Briggs institute (JBI) to assess their quality. The quality assessment tool includes 9 items. The specific content of each item is as follows: ①Does the sample represent the target population?②Is the sampling method appropriate for the study population? ③Is the sample size sufficient? (At least≥200 ) ④Are the study subjects and research settings described in detail? ⑤Do the subgroups in the sample have similar response rates to ensure sufficient coverage during data analysis? ⑥Is an effective method used to identify the health issue? ⑦Are standard and reliable methods used to assess the health issue in all study subjects? ⑧Is the data analysis method appropriate? ⑨Are the response rates sufficient? Are appropriate methods used to address low response rates?

The evaluation results are defined based on four dimensions: "yes," "no," "unclear," and "not applicable." A score of 1 is assigned for "yes," while all other responses receive a score of 0. The score below 5 is considered low-quality and excluded from the analysis (6, 7).

### 2.4 Literature exclusion

①The study population included patients with rubella, suspected rubella cases, or with additional symptoms or diseases; ②The timing and method of seroprevalence testing of the study population are unspecified; ③The types of literature are conference papers, clinical trial studies, animal experiments, discussions on the prevalence of the disease and so on; ④The objective of the literature is to explore the effectiveness of the vaccine itself, such as the antibody conversion rate; ⑤The literature has a low-quality assessment score.

### 2.5 Statistical analysis

The data analysis in this study utilized the "meta" package in R4.3.2 for conducting meta-analysis. The positive rate of the antibody served as the statistical analysis index, with the corresponding 95% confidence interval (CI) calculated according. We evaluate the heterogeneity among studies using the I^2^. If I^2^<50%, the heterogeneity among the studies was low, and a fixed-effects model was used for combining. Conversely, the random effects model was used. The Egger's test combined with a funnel plot was used to evaluate publication bias, and sensitivity analysis was conducted using a one-by-one exclusion approach. Two-sided tests will be performed with a significance level of α=0.05.

## 3 Results

### 3.1 Literature screening process

**Figure 1:**
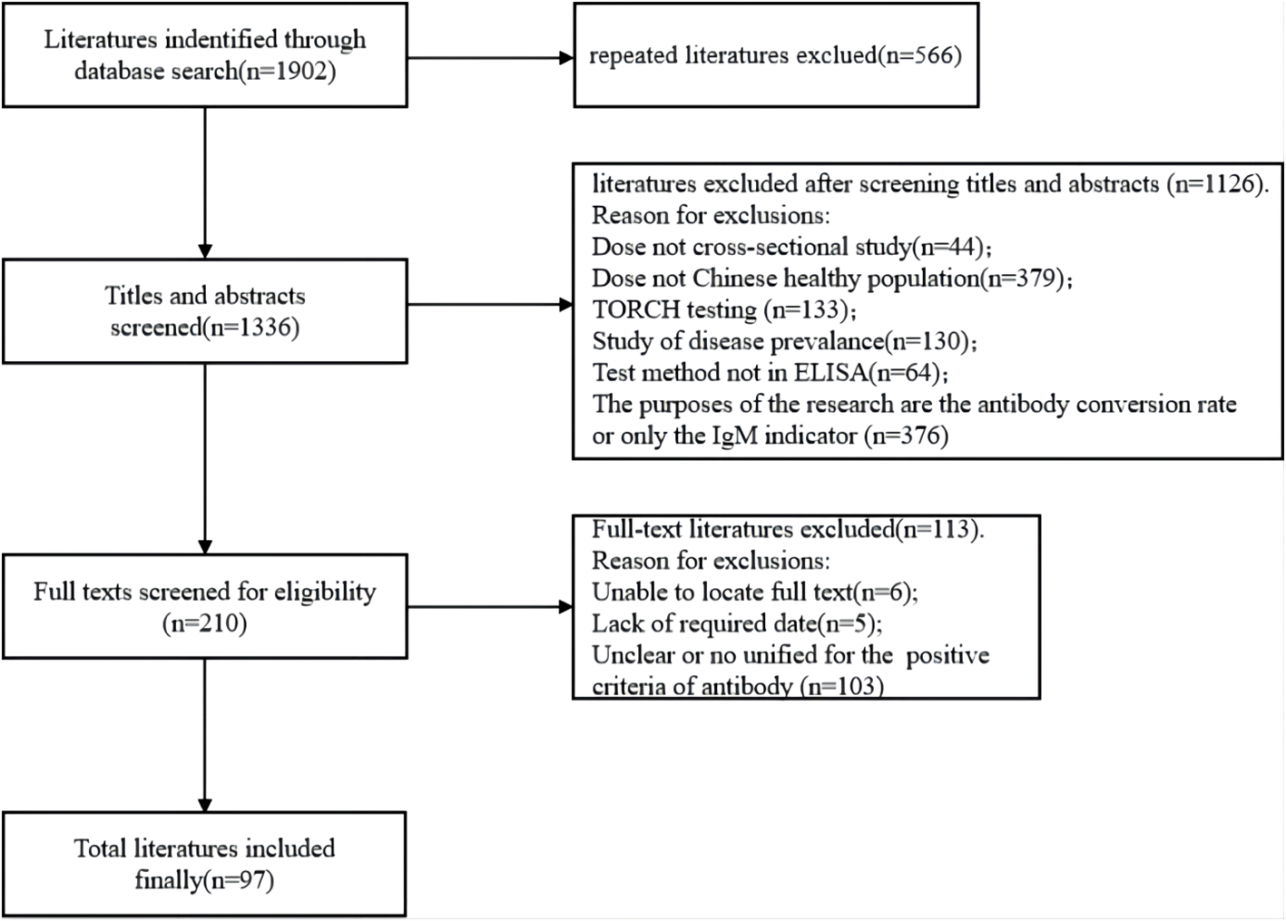
Flow chart of the literature screening.

After conducting an initial database search using the specified search terms, a total of 1902 articles identified. Around 566 articles were removed due to the duplication. Following the inclusion and exclusion criteria, 97 articles were ultimately selected (**Figure 1**). Among these, 94 were written in Chinese while 3 were in English.

### 3.2 Study characteristics

The 97 included papers are all cross-sectional studies published between 2003 and 2023, encompassing seven geographical regions in China. The maximum sample size of the literature was 11013 and the minimum sample size was 197. The literature quality scores ranged from 5 to 8, with the highest score being 8 and the lowest score being 5 (**Table 1**).

**Table 1:** Study characteristics.

Author	Province	The year of study	Subjects	Age (years)	Relevant factor*	Quality score
Akezhong et al. (2016) (8)	Qinghai	2014	364	<20	—	5
Cai ZK et al. (2003) (9)	Fujian	2001	342	1-19	a、b	6
Chen JH et al. (2009) (10)	Fujian	2007	1296	0-44	b、c	5
Cao H et al. (2014) (11)	Tianjin	—	210	0-24	a、d	5
Cao Y et al. (2017) (12)	Chongqing	2014	3230	2-15	b	6
Feng YF et al. (2021) (13)	Guangdong	2020	238	≥0	—	6
Wang HL et al. (2016) (14)	Zhejiang	2014	2491	0-60	—	7
Zhang H et al. (2009) (15)	Fujian	2007	477	0-44	a	6
Gong H et al. (2016) (16)	Sichuan	2016	224	≥0	a、c、d	6
Chen SJ et al. (2018) (17)	Sichuan	—	287	≥0	—	5
Chen YW et al. (2022) (18)	Fujian	2019	561	0-59	a、d	6
Li S et al. (2016) (19)	Jiangxi	2015	307	0-9	—	6
Cheng J et al. (2011) (20)	Hubei	2010	700	≥0	—	6
Deng YB et al. (2016) (21)	Xinjiang	2014	215	≥0	a	5
Xianling Wang et al. (2010) (22)	Tianjin	2007	608	0-57	—	6
Fan Y et al. (2020) (23)	Sichuan	2018	2107	≥0	a、d	5
Li L et al. (2022) (24)	Guangdong	—	389	≥0	d	7
Geng LN et al. (2017) (25)	Jiangsu	2012	500	0-40	d	5
Gu F et al. (2012) (26)	Henan	2010	363	2-25	—	6
Guan TJ et al. (2013) (27)	Guangdong	—	359	≥0	—	5
Guo W et al. (2013) (28)	Shandong	2011	328	≥0	—	5
Hao BY et al. (2023) (29)	Tianjin	2020	290	0-44	a、d	7
He PQ et al. (2016) (30)	Gansu	2015	585	0-49		6
He Q et al. (2023) (31)	Guangdong	2020	1099	≥0	a、b、c	8
Hu JYet al.(2008) (32)	Shanghai	2005	714	0-40	—	5
Hu YL et al. (2017) (33)	Henan	—	1155	—	—	6
Hu YG et al. (2018) (34)	Sichuan	2012、2016	428	≥0	—	6
Xing WY et al. (2018) (35)	Jiangsu、Guangxi	2010	420	0-15	—	5
Huang C et al. (2023) (36)	Guangxi	—	1322	0-69	a、d	6
Yang C et al. (2019) (37)	Guangxi	2017	322	0-20	—	6
Huang N et al. (2018) (38)	Guizhou	2016	240	≥0	d	6
Ye N et al. (2021) (39)	Sichuan	2018	210	≥0	a	6
Lan X et al. (2018) (40)	Sichuan	2015	681	≥0	a、b、c、d	6
Sheng WH et al. (2022) (41)	Guangdong	2020	240	≥0	—	7
Yang AX et al. (2016) (42)	Xinjiang	2015	239	0-45	—	6
Li JM et al. (2016) (43)	Xinjiang	2014	1056	0-59	a	6
Li JL et al. (2013) (44)	Shandong	2011	320	≥0	—	6
Li P et al. (2009) (45)	Shanxi	—	225	1-14	b	5
Li WX et al. (2012) (46)	Shandong	2010	661	2-14	—	6
Liu XL et al. (2020) (47)	Shanxi	2013	366	≥0	a、b	5
Ling X et al. (2020) (48)	Liaoning	2017	2430	≥0	d	6
Liu DS et al. (2018) (49)	Jiangsu	2016	314	0-40	—	6
Xu CH et al. (2020) (50)	Sichuan	2018	1901	≥0	—	5
Liu Y et al. (2020) (51)	Zhejiang	2016-2018	996	≥0	a、d	7
Liu Y et al. (2020) (52)	Chongqing	—	736	≥20	a	6
Liu Y et al. (2020) (53)	Chongqing	2018	2855	1-30	a	7
Liu Y et al. (2023) (54)	Sichuan	2022	1448	0-6	a、d	7
Lu HM et al. (2016) (55)	Shanghai	2013	360	0-40	a、c	6
Lv WY et al. (2017) (56)	Henan	2016	449	≥0	—	7
Lv WY et al. (2016) (57)	Henan	2015	420	≥0	—	7
Yang Y et al. (2016) (58)	Shanxi	2014	505	2-5	a、b、d	7
Wu MX et al. (2017) (59)	Fujian	2016	415	≥0	—	6
Ma Y et al. (2019) (60)	Shanxi	2017	960	0-50	b、d	7
Zhang FX et al. (2013) (61)	Shanxi	2011	280	≥0	—	6
Pang H et al. (2009) (62)	Shanghai	—	241	—	c	6
Qi QR et al. (2019) (63)	Henan	2018	2120	2-15	a	6
Wang AQ et al. (2013) (64)	Shandong	2011	320	≥0	—	5
Wang XB et al. (2014) (65)	Sichuan	2012	210	≥0	—	5
Ruan JW et al. (2023) (66)	Guangdong	2021	287	15-24	a、c	7
Sheng WH et al. (2015) (67)	Guangdong	2012	319	≥0	a、b	8
Shi ZH et al. (2016) (68)	Hebei	2014	197	0-19	—	5
Shu XD et al. (2016) (69)	Zhejiang	2015	290	≥0	a、c、d	7
Tang XM et al. (2013) (70)	Guizhou	2011	639	≥0	—	7
Tuerhong·musa et al. (2021) (71)	Xinjiang	2018	839	0-6	a、b、d	7
Wang CY et al. (2013) (72)	Shandong	2009-2010	4001	≥0	a	6
Wang J et al. (2023) (73)	Henan	2019-2021	996	0-79	d	8
Wang MC et al. (2010) (74)	Shandong	2009	245	≥0	—	5
Wang T et al. (2012) (75)	Beijing	2007	220	≥0	c	5
Wang WS et al. (2022) (76)	Liaoning	2020	498	≥0	a	6
Wang YL et al. (2012) (77)	Zhejiang	2009	384	≥0	—	6
Wu R et al. (2018) (78)	Xinjiang	2016	288	≥0	a	6
Hu Y et al. (2013) (79)	Jiangsu	2012	1502	≥0	—	5
Wu FY et al. (2021) (80)	Jiangxi	2015-2020	11013	≥0	—	8
Xing QM et al. (2016) (81)	Guangdong	2014	241	≥0	a	6
Xu Q et al. (2015) (82)	Shandong	2011	1599	15-24	a、c	6
Xuehelaiti·bayiding et al. (2018) (83)	Xinjiang	2016-2017	360	≥0	—	7
Zhu HM et al. (2015) (84)	Jiangsu	2013	500	≥0	—	5
Yan R et al. (2023) (85)	Zhejiang	2020	4455	0-59	a、c、d	7
Zhang RP et al. (2016) (86)	Chongqing	2014	2179	6-15	a、b	6
Yang JP et al. (2018) (87)	Shanghai	2014	363	≥0	—	6
Yang YN et al. (2023) (88)	Henan	2021	630	1-79	a、d	7
Wang J et al. (2016) (89)	Sichuan	2014	310	≥0	—	6
Yang YY et al. (2022) (90)	Shanghai	2010-2020	10828	≥0	—	6
Ye ZD et al. (2021) (91)	Sichuan	2018	349	0-15	a、d	8
Zhang AN et al. (2019) (92)	Jilin	2015-2016	3289	15-26	a、b、c	7
Zhang FX et al. (2011) (93)	Shanxi	2005	311	0-64	—	6
Zhang LW et al. (2009) (94)	Beijing	2007	220	0-61	d	6
Cheng JQ et al. (2010) (95)	Guangdong	2008	1003	0-60	a	6
Sun TT et al. (2023) (96)	Henan	2021	550	1-79	a、d	7
Zhu HL et al. (2016) (97)	Zhejiang	2011	242	≥20	a	6
Cheng H et al. (2016) (98)	Heilongjiang	2015	1381	—	—	5
Luo J et al. (2013) (99)	Guizhou	2011	210	≥0	a、d	6
Luo FJ et al. (2008) (100)	Beijing	—	303	≥0	—	5
Lai ZW et al. (2023) (101)	Hunan	2020-2022	2691	0-19	a、d	7
Lin WY et al. (2015) (102)	Guangdong	2012	1017	≥0	a、c	7
Wang J et al. (2022) (103)	Zhejiang	—	4362	≥0	a、b	7
Wang XZ et al. (2023) (104)	Zhejiang	—	778	≥0	a、b、d	7

*—represents the relevant data was not mentioned in the original literature. Among the relevant factors, "a" represents gender, "b" represents urban area, "c" represents household registration, and "d" represents immunization history.

### 3.3 Overall rubella seroprevalence

A total of 97 papers reported the seroprevalence of rubella in the population. The study encompassing 103,018 individuals, among whom 79,091 tested positive for antibodies. The highest positive rate was 98.20% while the lowest was 45.90%. Based on the random-effects model, the estimated positive rate of rubella antibody in the healthy Chinese population was 77.36% (95%CI:75.30-79.42,I^2^ = 98.50%).

### 3.4 Rubella seroprevalence in different age groups

A total of 96 papers were included in the analysis, reporting rubella seroprevalence across different ages groups. After integration of the data by age group that was previously set, the stratified comparison revealed a significant difference in the positive rate of rubella antibodies among different age groups (*χ^2^ =* 121.76,P<0.001). The<1-year-old group exhibited the lowest positive rate at 47.71% (95%CI:41.25-54.16), while the ≥50-years-old group showed the highest positive rate at 85.43% (95%CI:81.01-89.85) (**Table 2**).

**Table 2:** The analysis results of the positive rate of rubella antibodies in each subgroup.

Subgroup	No. of studies	Rubella positive	Total	95%*CI* (%)	Heterogenity	*χ^2^*	*P*
					*I^2^* (%)		
Age (y)							
<1	54	2696	7714	47.71 (41.25,54.16)	95.7	121.76	<0.001
1-4	69	13699	15809	85.26 (82.21,88.30)	93.9		
5-9	70	8473	11124	78.34 (75.17,81.51)	94.2		
10-14	33	4033	5759	74.00 (66.78,81.22)	98		
15-19	59	9119	11947	76.47 (71.68,81.27)	96.2		
20-34	32	6941	8586	81.43 (78.54,84.32)	88.8		
35-49	20	1614	2045	82.55 (78.67,86.44)	83.2		
≥50	18	1287	1572	85.43 (81.01,89.85)	89.5		
Gender							
Male	46	20689	25912	78.28 (74.95,81.62)	97.7	0.15	0.7
Female	46	19490	24228	79.18 (76.10,82.26)	97.1		
District							
Urban	15	7606	9162	79.50 (73.49,85.50)	95.6	0.1	0.757
Rural	15	9367	11420	78.12 (71.75,84.49)	95.8		
Registration							
permanent population	13	8581	10868	79.45 (73.25,85.64)	98.7	0.24	0.63
migrant population	13	3245	4115	77.52 (72.87,82.17)	90.2		
Regions of China							
East China	24	28981	38673	77.68 (73.88,81.48)	98.8	6.62	0.358
Southwest	18	14131	18244	74.01 (68.75,79.27)	98.9		
Northwest	14	5444	6593	80.40 (74.76,86.03)	95.2		
South China	17	7790	9927	76.53 (71.32,81.73)	96.3		
North China	7	2082	2804	77.16 (71.79,82.54)	91.3		
Central China	12	14700	19515	79.13 (72.88,85.38)	99.4		
Northeast	4	6181	7598	82.52 (77.55,87.48)	96.4		
Immunization history							
0dose	21	1744	3002	58.23 (47.20,69.25)	98.6	15.44	0.002
1dose	25	3068	3945	77.46 (73.01,81.92)	90.6		
≥2dose	24	7922	9994	80.45 (76.36,84.55)	96.3		
Unknown	12	3698	4723	80.61 (77.23,84.00)	82.8		
The year of study							
2001-2008	9	3709	5191	70.42 (62.17,78.68)	97.3	3.26	0.35
2009-2013	28	14808	18652	78.17 (74.28,82.25)	97.1		
2014-2018	47	37413	48737	75.15 (71.79,78.51)	98.7		
2019-2022	21	14581	20077	75.21 (70.38,80.04)	99.1		

### 3.5 Rubella seroprevalence in different gender groups

A total of 46 papers reported the seroprevalence of rubella across genders. The analysis revealed the positive rate of rubella antibody of 78.28% (95%CI:74.95-81.62) in males and 79.18% (95%CI:76.10-82.26) in females. There was no significant difference observed in the positive rate of rubella antibodies between the different gender groups (*χ^2^ =* 0.15,P=0.70) (**Table 2**).

### 3.6 Rubella seroprevalence in different district groups

A total of 15 papers reported the seroprevalence of rubella in different districts. The results showed that the positive rate of rubella antibody was 79.50% (95%CI:73.49-85.50) in the urban population and 78.12% (95%CI:71.75-84.49) in the rural population. There was no significant difference in the positive rate of rubella antibodies in different district groups (*χ^2^ =* 0.10, P=0.76) (**Table 2**).

### 3.7 Rubella seroprevalence in different registration groups

A total of 13 papers reported the seroprevalence of rubella in different registrations. The results showed that the positive rate of rubella antibody was 79.45% (95%CI:73.25-85.64) in permanent population and 78.12% (95%CI:71.75-84.49) in migrant population. No significant difference was found in the positive rates of rubella antibodies between the different registration groups (*χ^2^ =* 0.24, P=0.63) (**Table 2**).

### 3.8 Rubella seroprevalence among different regions of China

The Chinese provinces were categorized into seven regions according to their geographical location. The analysis revealed no significant difference in the positive rate of rubella antibody among different regions (*χ^2^ =* 6.99,P=0.32). The lowest positive rate of rubella antibody was 74.01%(95%CI:68.75-79.27) in the southwest region, while the highest positive rate was 82.52%(95%CI:77.55-87.48) in the northeast region (**Table 2**).

### 3.9 Rubella seroprevalence among groups with different immunization history

There was a significant difference observed among the different immunization history groups (*χ^2^ =* 8.2, P=0.02). The lowest positive rate of rubella antibody was 58.23% (95%CI:47.2-69.25) in the population with no RCV vaccination history, and the highest positive rate was 80.45% (95%CI:76.36-84.55) in the population with histories of receiving at least 2 doses of RCV (**Table 2**).

### 3.10 Rubella seroprevalence among different the year of study

After integration of the data by the groups of study year that previously set, the results of integration showed that the lowest rubella antibody positive rate was 70.42% (95%CI:62.17-79.56) during 2001-2008, while the highest value was 78.17% (95%CI:74.28-82.05) during 2009-2013. There was no significant difference in the positive rate of rubella antibodies among different registration groups (*χ^2^ =* 3.26,P=0.35) (**Table 2**).

### 3.11 Sensitivity analysis and publication bias

**Figure 2:**
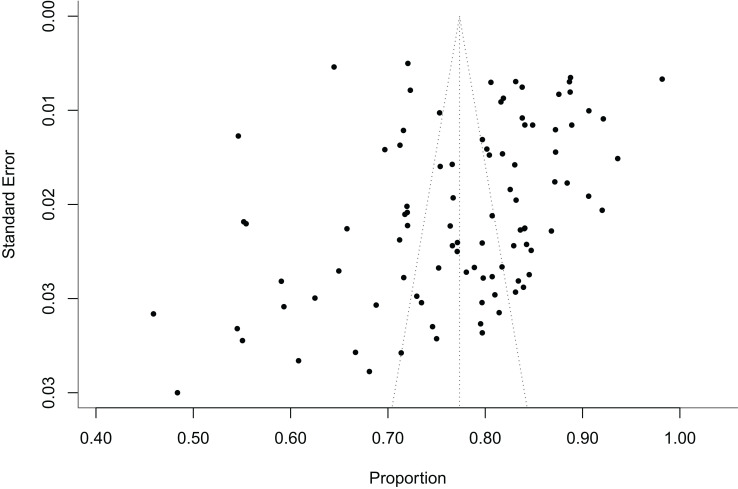
The funnel plots assessing the publication bias in the study of rubella antibody levels in the healthy Chinese population.

By conducting sensitivity analysis involving the stepwise exclusion of included studies, the results remained consistent with the original findings, indicating the stability of the meta-analysis results. The publication bias was not found in this Meta-analysis, which was confirmed by using Egger's test (P=0.08) and shown in **Figure 2**. (Each point represents the included article).

## 4 Discussion

In 2012, WHO established the goal of rubella elimination as part of the "Global Measles and Rubella Strategic Framework 2012-2020". In 2015, the WHO Region of the Americas verified that 35 countries and regions within that region were eliminated rubella. The World Health Organization's Western Pacific Region (WPRO), to which China belongs, set a goal for rubella elimination in 2014 (3). As of 2021, several WPRO member countries and regions, including Australia, Brunei, New Zealand, South Korea, Macau, and Hong Kong, have declared rubella elimination (105, 106). In the “Global Measles and rubella Strategic Framework 2021-2030” issued by WHO in 2021, it is pointed out that strengthening the control and elimination of rubella can be combined with measles. The framework aims to achieve and maintain regional elimination of measles and rubella by 2030 (107). While seroepidemiological survey data can reflect the epidemiological characteristics of rubella, routine monitoring of rubella seroprevalence has not been conducted in China. It is resulting in a lack of comprehensive and dynamic control and understanding. This paper conducted a meta-analysis to explore the level of rubella antibody in healthy people in China from the whole to the extraction of relevant influencing factors.

The WHO has indicated that achieving the rubella seroprevalence of over 83% to 85% is necessary to establish robust herd immunity (80). Our study showed that the rubella seroprevalence in healthy people in China was 77.36%, lower than the standard recommended by WHO. Weak immunity threshold levels can lead to an increased incidence of rubella in high-risk populations and an increased risk of CRS in children.

To enhance rubella prevention and control efforts, RCV was incorporated into the immunization program in China in 2008, and the positive rate of rubella antibody has increased significantly since 2009. From this study, it can be seen that when there was no large-scale introduction of RCV, the rubella seroprevalence can be as low as 45.9% (9). The introduction of the vaccine has significantly increased the rubella seroprevalence in the population. It was found that the rubella seroprevalence increased with the increase in vaccination dose, which was consistent with the results of Wang Jun et al. (73). Similarly, a study in Beijing on the detection of measles and rubella seroprevalence in children aged 18-24 months after multiple doses of the MMR vaccine found that the antibody positive rate and antibody Geometric mean concentration (GMC) increased significantly after multiple doses. The positive rate of rubella antibody increased from 97.22% to 100%, and the GMC reached (283.52 ± 90.83)IU/ml (108). Although a single dose of MMR can obtain more than 95% long-term immunity (109), the annual decline rate of seroprevalence of a single dose vaccine is 0.014 (0.012-0.017), showing an exponential attenuation. After receiving two doses of MMR, the decline rate of seroprevalence decreased to 0.012 (0.010-0.014) (110). Multiple doses of vaccine, such as two or more, can enhance the seroprevalence and promote immune persistence in healthy individuals.

Several factors influence the level of rubella antibody. Based on the findings of this study, age and vaccination history emerge as significant factors that impact the level of rubella antibody. The modified immunization schedule has significantly contributed to the variations in rubella seroprevalence across different age groups.

The rubella immunization schedule in China involves administering the RCV at 8 months and 18 months of ages, respectively (111). The majority of children aged 1-4 years have a history of RCV, leading to a higher positive rate of antibodies. Conversely, children below 8 months of age have not yet reached the age for RCV and depend on maternally-derived antibodies. Additionally, Zhu Q et al. showed that newborn measles and rubella antibody levels increased with the increase of maternal antibody levels (118). However, since the mothers of infants included in this analysis were born in the period when rubella vaccine was not widely administered, fewer were vaccinated, most of which were immune after tacit infection with rubella virus (119). Consequently, the level of maternal antibodies has decreased, which is associated with the low antibody positivity rate observed in children under 1-year-old. Since the current RCV program primarily focuses on children, the positive rate of rubella antibody starts to decrease after the age of 5. Studies have shown that individual antibody levels decrease over time in the absence of widespread virus transmission (121). This finding may be one of the reasons why antibody levels drop after age of 5. The relevant studies have demonstrated that the risk of rubella will increase with time (112), and the use of rubella vaccine in children cannot reduce the incidence of rubella in adolescents and adults (113). Currently, the main affected population for rubella in China is concentrated among middle school students (3), and in the outbreak of rubella in 2018-2019, the age of onset was concentrated in the 10-29 age group (105). In response to this phenomenon, Zhejiang Province implemented intensified RCV immunization for the third-year middle school students. After enhanced immunization of 15-19 age group, the positive rate of rubella antibody and GMC were significantly higher than those of 10-14 age group (114). Therefore, administering RCV to rubella-susceptible populations, such as adolescents and adults who failed to respond to the primary vaccination, can compensate for the immunological gap created by basic childhood immunity (3, 117). This could prevent and control adult rubella epidemics (114). Individuals aged 35 and above, who were born before the inclusion of RCV in the national expanded immunization program have had more opportunities for exposure to infectious sources, which has led to higher levels of naturally acquired antibodies. According to the data of a study on the introduction of RCV in the immunization program, the annual incidence of rubella decreased from 91.09 cases per million to 3.31 cases per million after the vaccine was widely used. This means that most people at that time had a higher risk of exposure to rubella, which may be one of the reasons for the high positive rate of rubella in older age groups (120). It may be one of the reasons for the high positive rate of rubella in older age groups.

There was no statistically significant difference in rubella seroprevalence between genders, which is consistent with the majority of findings in the Chinese literature (29, 71, 94). Our study with age-specific subgroup analysis revealed a relatively low prevalence of rubella seropositivity among women of reproductive age. It may lead to an increased risk of CRS infection, and there is a need to strengthen the immunity threshold levels in this age group (115). The reduction of CRS occurrence in the United States was achieved by recommending one dose of MMR for adults, based on the implementation of two doses of MMR for children (115). Similarly, a 75% reduction in the number of cases of CRS in schoolgirls vaccinated with the RCV was achieved in the United Kingdom (116). These findings suggest that women of childbearing age who are either unvaccinated or do not have sufficient evidence of rubella-specific immunity may benefit from receiving the RCV to boost their seropositive level.

Our study has several limitations. First the included studies were predominantly cross-sectional, which are susceptible to measurement bias and non-response bias. Second, some primary studies lacked adequate analysis of study factors, had small sample sizes, or focused on narrow age groups, contributing to increased heterogeneity in the literature. Third, our study adopts the commonly used definition in the literature of antibody positive rate, which is more than 20 IU/ml. However, there are variations in definitions across studies, with some using a threshold of more than 10 IU/ml or lacking a clear definition altogether, potentially leading to non-unique meta-analysis results. In view of these issues, we adopted a strategy of expanding the number of papers and broadening the coverage of time and geographical dimensions to minimize the impact on the results and uphold scientific rigor to a certain extent.

## 5 Conclusion

In conclusion, to achieve the goal of rubella elimination, China needs to implement a comprehensive strategy that integrates rubella disease surveillance and serological testing. This approach should be built upon a high vaccination coverage rate for RCV within the immunization program. Furthermore, it is crucial to timely targeted immunization efforts for high-risk groups, such as middle school students and women of childbearing age, to enhance their immunity against rubella. By establishing a robust rubella immunity threshold levels, significant reductions can be achieved in the harm caused by rubella and its associated complications.

## Data Availability

The datasets presented in this article are not readily available because you need to send an email request before using this data. Requests to access the datasets should be directed to smileforever81@126.com.
